# Human RIPK3 C-lobe phosphorylation is essential for necroptotic signaling

**DOI:** 10.1038/s41419-022-05009-y

**Published:** 2022-06-23

**Authors:** Yanxiang Meng, Christopher R. Horne, Andre L. Samson, Laura F. Dagley, Samuel N. Young, Jarrod J. Sandow, Peter E. Czabotar, James M. Murphy

**Affiliations:** 1 Walter and Eliza Hall Institute of Medical Research, 1G Royal Parade, Parkville, VIC 3052 Australia; 2 Department of Medical Biology, University of Melbourne, Parkville, VIC 3052 Australia

**Keywords:** Kinases, Necroptosis

## Abstract

Necroptosis is a caspase-independent, pro-inflammatory mode of programmed cell death which relies on the activation of the terminal effector, MLKL, by the upstream protein kinase RIPK3. To mediate necroptosis, RIPK3 must stably interact with, and phosphorylate the pseudokinase domain of MLKL, although the precise molecular cues that provoke RIPK3 necroptotic signaling are incompletely understood. The recent finding that RIPK3 S227 phosphorylation and the occurrence of a stable RIPK3:MLKL complex in human cells prior to exposure to a necroptosis stimulus raises the possibility that additional, as-yet-unidentified phosphorylation events activate RIPK3 upon initiation of necroptosis signaling. Here, we sought to identify phosphorylation sites of RIPK3 and dissect their regulatory functions. Phosphoproteomics identified 21 phosphorylation sites in HT29 cells overexpressing human RIPK3. By comparing cells expressing wild-type and kinase-inactive D142N RIPK3, autophosphorylation sites and substrates of other cellular kinases were distinguished. Of these 21 phosphosites, mutational analyses identified only pT224 and pS227 as crucial, synergistic sites for stable interaction with MLKL to promote necroptosis, while the recently reported activation loop phosphorylation at S164/T165 negatively regulate the kinase activity of RIPK3. Despite being able to phosphorylate MLKL to a similar or higher extent than wild-type RIPK3, mutation of T224, S227, or the RHIM in RIPK3 attenuated necroptosis. This finding highlights the stable recruitment of human MLKL by RIPK3 to the necrosome as an essential checkpoint in necroptosis signaling, which is independent from and precedes the phosphorylation of MLKL.

## Introduction

Necroptosis is a lytic, caspase-independent, pro-inflammatory form of programmed cell death [1–4]. The terminal effector of necroptosis, mixed lineage kinase domain-like (MLKL), executes cell death by permeabilizing the plasma membrane and spilling cellular contents, which act as damage-associated molecular patterns (DAMPs) to elicit inflammatory responses [2, 5, 6]. Consistent with its pro-inflammatory nature, necroptosis likely has evolved as an innate immune mechanism to eliminate pathogens under conditions when apoptosis is blocked [7–9]. However, recent studies have implicated the dysregulation of necroptosis in a variety of human pathologies, such as inflammatory diseases [10–12], inflammatory bowel disease [13], metabolic diseases [14], and ischemia-reperfusion injury [15–17]. Consequently, the necroptosis pathway has attracted much attention as a potential therapeutic target for the treatment of various infectious and inflammatory conditions. As such, mechanistic knowledge about the signaling cascade that leads to cell death by necroptosis is of enormous interest.

Necroptosis can be induced by the activation of TNF receptors [1], or various pattern recognition receptors such as Toll-like receptors [18] or nucleic acid sensors [19]. Amongst these, the necroptosis pathway downstream of TNFR1 activation by TNF is the most well-studied. Under conditions when the cellular inhibitors of apoptosis proteins (cIAP) family of E3 ubiquitin ligases and the pro-apoptotic Caspase-8 are both inhibited or depleted, receptor-interacting serine/threonine protein kinase (RIPK)-1 and RIPK3 assemble into a high molecular weight signaling platform known as the necrosome. This is mediated by the formation of heteroamyloid fibrils involving the RIP homotypic interaction motifs (RHIM) that reside C-terminal to the kinase domains of RIPK1 and RIPK3 [20, 21]. Subsequently, MLKL is phosphorylated by the kinase domain of RIPK3 [2, 22], which is considered the hallmark of necroptosis activation. MLKL phosphorylation prompts conformational switching [23–25] and assembly into active oligomers [26], before trafficking via Actin-, Golgi-, and microtubule-dependent mechanisms to the plasma membrane [27] where it disrupts the membrane and causes cell death [28, 29].

The precise mechanism of necroptosis signaling remains incompletely understood. As we recently reviewed, the necroptosis pathway is heavily regulated by post-translational modifications [30]. The autophosphorylation (S166) of RIPK1 has been proposed to be a prerequisite for necrosome formation [31–34], while the autophosphorylation of human RIPK3 at S227 (T231/S232 in mouse RIPK3 [35, 36]) is required to recruit MLKL to the necrosome [2]. Elevated RIPK3 S227 phosphorylation (pS227) was observed after necroptosis stimulation [2], leading to the idea that pS227 is the cue for RIPK3 activation and downstream MLKL recruitment and phosphorylation. Recently, structural studies of human and mouse RIPK3:MLKL complexes have shed light on the species-specific recognition mechanism and highlighted the role of RIPK3:MLKL stable pre-association at steady state for necroptosis signaling in human cells [23, 37]. RIPK3 pS227 is required for RIPK3:MLKL stable interaction, and RIPK3:MLKL stable interaction can be detected endogenously in human cells before necroptosis is induced, indicating RIPK3 pS227 must already exist under basal conditions. This raises the question of what cues beyond pS227 are required to provoke RIPK3 necroptotic signaling, and whether an unknown (auto)phosphorylation event activates RIPK3 upon exposure to necroptosis signals.

Here, we sought to identify (auto)phosphorylation sites of human RIPK3 using phosphoproteomics. By overexpressing human RIPK3, an abundance of phosphopeptides was obtained, from which 21 unique RIPK3 phosphorylation sites were identified. By comparing cells expressing wild-type RIPK3 with that carrying the kinase-inactive D142N mutation, we identified autophosphorylation sites as well as the substrates of other cellular kinases. Extensive mutagenesis analyses revealed that phosphorylation of only two of the 21 RIPK3 sites identified, T224 and S227, were crucial for necroptotic signaling, while the recently reported phosphorylation of activation loop residues, S164/T165 [38], negatively regulated RIPK3 kinase activity. Despite being able to phosphorylate MLKL to a similar or even higher extent than the wild-type RIPK3, T224, S227, or RHIM mutations in RIPK3 attenuated or abolished their abilities to mediate necroptosis. Our findings highlight the stable recruitment of human MLKL to the necrosome as an essential checkpoint in necroptosis signaling, which is required in addition to MLKL phosphorylation for the execution of necroptotic cell death.

## Methods

### Expression constructs

Genes encoding wild-type or mutant human RIPK3 with a N-terminal FLAG tag were either synthesized by ATUM or generated by oligonucleotide-directed overlap PCR from a human RIPK3 DNA template (ATUM) and ligated into pF TRE3G PGK puro [4] as BamHI-EcoRI fragments. Insert sequences were verified by Sanger sequencing (Australian Genome Research Facility, VIC, Australia). Lentiviral particles were generated by co-transfecting vector DNA with pVSVg and pCMV ΔR8.2 helper plasmids into HEK293T cells. *RIPK3*^−*/*−^ HT29 cells [39] were transduced and selected for genomic integration using puromycin (2.5 μg/mL; StemCell Technologies) using established procedures [4, 26, 40].

### Reagents and antibodies

Primary antibodies used in this study were: rat anti-human RIPK3 (clone 1H2, produced in-house; 1:1000; available as MABC1640, EMD Millipore, Billerica, MA, USA) [25], rat anti-mouse RIPK3 (clone 1H12, produced in-house; 1:1000) [41], rabbit anti-human RIPK3 phospho-S227 (D6W2T, CST, 1:2000), rabbit anti-human RIPK3 phospho-S227 (EPR9627, Abcam; 1:2000) rat anti-human MLKL pseudokinase domain (clone 7G2, produced in-house; 1:2000 dilution; available as MABC1636, EMD Millipore, Billerica, MA, USA) [27], rat anti-human MLKL (3H1, ﻿produced in-house; 1:1000; available as MABC604, EMD Millipore, Billerica, MA, USA) [25], mouse anti-human RIPK1 (610459, BD Transduction Laboratories; 1:1000), rabbit anti-human MLKL phospho-S358 (AB187091, Abcam; 1:2000), mouse anti-GAPDH (MAB374, Millipore; 1:2000) and anti-Actin (C4) HRP (sc-47778 HRP, Santa Cruz Biotechnology; 1:10,000). The Smac mimetic, Compound A [42] and the pan-caspase inhibitor, IDN-6556/Emricasan, were provided by Tetralogic Pharmaceuticals, and recombinant hTNF-Fc [43] was produced in-house.

### Cell culture

Human DMEM (ThermoFisher) media supplemented with 8% v/v fetal calf serum (FCS; Sigma) was used to culture the human colorectal adenocarcinoma HT29 (available from ATCC) and their *RIPK3*^−*/−*^ counterpart [39]. Puromycin (2.5 μg/mL; StemCell Technologies) was added for lines stably transduced with doxycycline inducible RIPK3 constructs. Routine PCR testing confirmed cell lines to be mycoplasma-negative.

### IncuCyte cell death assay

HT29 cells were seeded into 48-well plates at 3 × 10^4^ cells/well. After settling for 16–24 h, cells were treated with doxycycline (2.5 ng/mL) overnight to induce expression of the relevant RIPK3 construct, before being treated with TNF (100 ng/mL), the Smac-mimetic compound A (500 nM) and the pan-caspase inhibitor IDN-6556 (5 μM) in FluoroBrite DMEM (Thermo Fisher Scientific) media supplemented with 1% v/v FCS (Sigma), 1 mM Na pyruvate (Thermo Fisher Scientific), 1 mM l-GlutaMAX, 500 nM SYTOX Green (Thermo Fisher Scientific), and 1 μM DRAQ5 fluorescent probe (Thermo Fisher Scientific) to induce necroptosis. Cells were imaged on ×10 objective in an IncuCyte SX5 System (Essen Bioscience) using the default bright-field channel, green channel, and near infrared (NIR) channel settings with scans every hour for 24 h. The number of SYTOX Green-positive cells per mm^2^ over time was quantified and normalized by the number of DRAQ5-positive cells per mm^2^ to measure percent cell death using the IncuCyte SX5 v2021B software (Sartorius).

### Western Blots

*RIPK3*^−*/*−^ HT29 cells reconstituted with FLAG-RIPK3 were seeded into 24-well plates at 7 × 10^4^ cells/well and left to settle overnight, before being treated with 2.5 ng/mL doxycycline to induce RIPK3 expression. Cells were then treated with TNF (100 ng/mL), the Smac-mimetic compound A (500 nM) and the pan-caspase inhibitor IDN-6556 (5 μM) to induce necroptosis in DMEM media supplemented with 1% FCS, penicillin (100 U/mL) and streptomycin (100 μg/mL). Cells were harvested 7.5 h post necroptotic induction (with TSI) in 2X SDS Laemmli lysis buffer (126 mM Tris–HCl, pH 8, 20% v/v glycerol, 4% w/v SDS, 0.02% w/v bromophenol blue, 5% v/v 2-mercaptoethanol), boiled at 100 °C for 5–10 min, and resolved on 4–15% Tris–Glycine gels (Bio-Rad). Proteins were transferred to polyvinylidene difluoride (PVDF; Merck, #IPFL00010) membranes and probed with antibodies as indicated. HT29 cells, and their *RIPK1*^−*/*−^, *RIPK3*^−*/*−^, or *MLKL*^−*/*−^ counterparts were treated with extrinsic apoptotic stimulant (TS; TNF and Smac-mimetic Compound A), necroptotic stimulant (TSI; TNF, Smac-mimetic Compound A, and IDN-6556), or intrinsic apoptotic stimulant (A + M; A, 1 μM ABT-737, Abbott Laboratories; M, 0.1 μM Mcl-1 inhibitor S63485, custom synthesized by SYNthesis MedChem) for 7.5 h, before being lysed in ice-cold RIPA buffer (10 mM Tris–HCl pH 8.0, 1 mM EGTA, 2 mM MgCl_2_, 0.5% v/v Triton X-100, 0.1% w/v Na deoxycholate, 0.5% w/v SDS and 90 mM NaCl) supplemented with 1× Protease & Phosphatase Inhibitor Cocktail (Cell Signaling Technology #5872S) and 100 U/mL Benzonase (Sigma #E1014). Whole-cell lysates were boiled for 10 min in 1× SDS Laemmli sample buffer, resolved by 1.5 mm NuPAGE 4–12% Bis–Tris gel (ThermoFisher Scientific #NP0335BOX) using MES Running buffer (ThermoFisher Scientific #NP000202), and transferred to PVDF membranes. Membranes were either blocked in Odyssey Blocking Buffer and probed with primary antibodies indicated, then the appropriate IR-dye-conjugated secondary antibodies, and signals were detected with an Odyssey CLx Imaging System (LI-COR); or blocked in 5% w/v skim milk powder in TBS-T, probed with primary antibodies then the appropriate HRP-conjugated secondary antibody and signals revealed by enhanced chemiluminescence (Merck P90720) on a ChemiDoc Touch Imaging System (Bio-Rad). Uncropped western blots are included as Supplemental Data.

### Immunoprecipitation for mass spectrometry

HT29 *RIPK3*^−*/*−^ cells reconstituted with either wild-type FLAG-RIPK3 or a D142N mutant were seeded into 15 cm plates at 1 × 10^7^ cells/plate and left to attach overnight. On day 2, cells were treated with 500 ng/mL doxycycline overnight, before being treated with TNF (100 ng/mL), the Smac-mimetic compound A (500 nM) and the pan-caspase inhibitor IDN-6556 (5 μM) to induce necroptosis for 3 h. Cells were harvested in lysis buffer (50 mM Tris–HCl pH 7.4, 1% v/v Triton X-100, 150 mM NaCl, 1 mM EDTA, 1 mM PMSF, 2 mM sodium vanadate, 10 mM sodium fluoride, Complete protease inhibitor tablet (Roche)) and supernatants divided equally into two fractions. One fraction was spiked with 50 μg/mL FLAG peptide as a negative control. Each cell lysate was mixed with 15 μL anti-FLAG M2 Affinity Gel (Millipore) and allowed to bind for 1 h at 4 °C with gentle agitation. ﻿Beads were washed in lysis buffer 3 times, before performing two elutions with 50 μL of 0.5 mg/mL FLAG peptide in Dulbecco’s phosphate-buffered saline (Thermo Fisher Scientific). Eluates were stored at −80 °C until further processing. Five replicates were performed in total.

### Recombinant protein preparation for mass spectrometry

Recombinant human MLKL pseudokinase domain (residues 190–471), human RIPK3 kinase domain (residues 1–316):MLKL pseudokinase domain (residues 190–471) complex, and human RIPK3 kinase domain (residues 2–316), were expressed and purified from *Sf*21 insect cells as previously described [24, 37, 44] using bacmids derived from the pFastBac Htb vector, which encodes an N-terminally fused TEV protease-cleavable His_6_ tag. After completing purification of proteins and determining their concentrations by measuring absorbance at 280 nm, 20 μg purified RIPK3:MLKL complex, 10 μg RIPK3 kinase domain, and 10 μg MLKL pseudokinase domain were subjected to further processing by tryptic digest and filter-aided sample preparation.

### Sample digestion and analysis by mass spectrometry

Protein samples were resuspended in 6 M Urea, 10 mM TCEP and 100 mM Tris–HCl pH 7.0 and subjected to protein digestion using FASP (filter aided sample preparation) before lyophilisation to dryness using a SpeedVac AES 1010 (Savant, ThermoFisher) [45]. Samples were analyzed on a nanoElute UHPLC (plug-in V1.0.10.4; Bruker, Germany) coupled to a timsTOF Pro (Bruker) mass spectrometer equipped with a CaptiveSpray source. Peptides were resuspended in 2% ACN, 1% formic acid (FA) separated on a 25 cm ×75 μm analytical column, 1.6 μm C18 beads with a packed emitter tip (IonOpticks, Australia). The column temperature was maintained at 50 °C using an integrated column oven (Sonation GmbH, Germany). The column was equilibrated using 4 column volumes before loading each sample in 100% buffer A (99.9% MilliQ water, 0.1% FA) (both steps were performed at 980 bar). Samples were separated at 400 nl/min using a gradient from 2% to 17% buffer B (99.9% ACN, 0.1% FA; 55 min), 17% to 25% buffer B (21 min) before ramping to 35% buffer B (13 min), ramp to 85% buffer B (3 min) and sustained for 10 min. The timsTOF Pro (Bruker) was operated in PASEF mode using Compass Hystar 5.0.36.0. Settings were as follows: mass range 100–1700*m*/*z*, 1/K0 Start 0.6 V s/cm^2^ End 1.6 V s/cm^2^, ramp time 109.9 ms, lock duty cycle to 100%, capillary voltage 1600 V, dry gas 3 l/min, dry temperature 180 °C, PASEF settings: 10 MS/MS scans (total cycle time 1.26 s), charge range 0–5, active exclusion for 0.4 min, scheduling target intensity 20,000, intensity threshold 2500, CID collision energy 42 eV.

All raw files were analyzed by MaxQuant v1.6.15.0 software using the integrated Andromeda search engine. Experiment type was set as TIMS-DDA with no modification to default settings. Data was searched against the human Uniprot Reference Proteome with isoforms (downloaded March 2019) and a separate reverse decoy database using a strict trypsin specificity allowing up to 2 missed cleavages. The minimum required peptide length was set to 7 amino acids. Modifications: carbamidomethylation of Cys was set as a fixed modification, while N-terminal acetylation of proteins, oxidation of Met and phosphorylation of Ser, Thr, and Tyr were set as variable modifications. First search peptide tolerance was set at 20 ppm and main search set at 10 ppm (other settings left as default). Matching between runs and LFQ quantitation was turned on. Maximum peptide mass [Da] was set at 8000. All other settings in group or global parameters were left as default.

Further analysis was performed using a custom pipeline developed in R (3.6.1), which utilizes the LFQ intensity values in the MaxQuant output file proteinGroups.txt. Proteins not found in at least 50% of the replicates in one group were removed. Missing values were imputed using a random normal distribution of values with the mean set at mean of the real distribution of values minus 1.8 s.d., and a s.d. of 0.3 times the s.d. of the distribution of the measured intensities. The probability of differential site modification expression between groups was calculated using the Limma R package (3.4.2).

## Results

### Enrichment of human RIPK3 via FLAG-immunoprecipitation for mass spectrometry

**Fig. 1:**
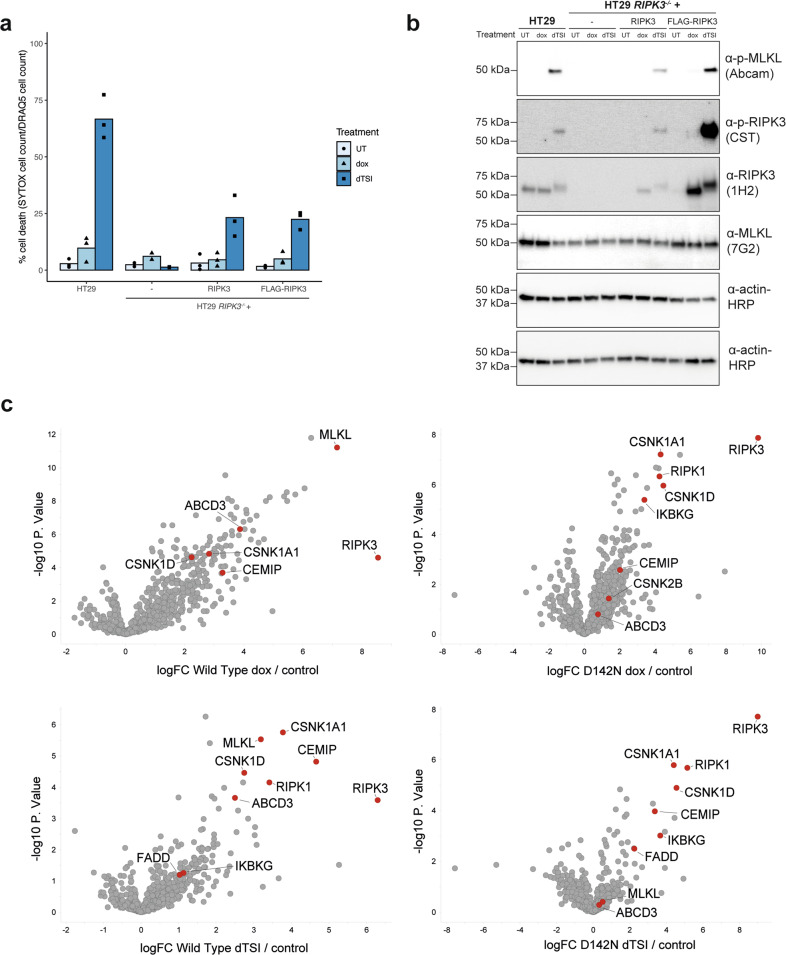
Enrichment of human RIPK3 from HT29 cells via FLAG-immunoprecipitation for mass spectrometry. **a** HT29 *RIPK3*^−/−^ cells harboring a doxycycline-inducible FLAG-RIPK3 construct reconstituted necroptosis to a similar level to a human RIPK3 construct without a FLAG tag. Cell death (quantified by SYTOX Green uptake) was measured as a percentage of total number of cells (quantified by DRAQ5 uptake) using IncuCyte imaging after treatment with necroptotic stimuli, dTSI (d, doxycycline; T, TNF; S, Smac mimetic Compound A; I, pan-caspase inhibitor IDN-6556), for 24 h. The means of three independent experiments (*n* = 3) are displayed as bars. Values of each independent experiment are shown as points. **b** HT29 *RIPK3*^−*/*−^ cells harboring a doxycycline-inducible FLAG-RIPK3 construct phosphorylated MLKL to a similar extent to endogenous RIPK3 in HT29 cells by immunoblotting. Following 2.5 ng/mL doxycycline induction overnight, cells were treated with dTSI for 7.5 h before lysis and immunoblotting. **c** Volcano plots of the log_2_-based ratio of peptide abundance for proteins between dox (top; 500 ng/mL doxycycline overnight) or dTSI (bottom; 500 ng/mL doxycycline overnight, followed by TSI for 3 h) conditions and the untreated control condition. Cell lysates from HT29 *RIPK3*^−*/*−^ cells overexpressing doxycycline-inducible FLAG-RIPK3 were harvested and subjected to FLAG-immunoprecipitation, before tryptic digest and analysis by mass spectrometry. Data from five biological replicates (*n* = 5). Proteins of interest are colored in red and labeled.

To identify human RIPK3 interacting proteins and sites of phosphorylation upon engagement of necroptotic signaling, we used lentiviral vectors to stably introduce doxycycline-inducible wild-type (WT) or kinase-inactive D142N [37] mutant human RIPK3 constructs into HT29 human colorectal adenocarcinoma cells from which *RIPK3* was deleted by CRISPR-Cas9 editing (Supplementary Fig. 1). RIPK3 expression was induced by doxycycline, before cells were treated with the necroptotic stimulus, TSI (TNF; Smac mimetic Compound A; pan-Caspase inhibitor IDN-6556/Emricasan), for 3 h to provoke cell death. Cell death was monitored using IncuCyte live cell imaging, where dead cells were stained with SYTOX Green, and total cells were stained with DRAQ5 (Fig. 1a). As expected, wild-type RIPK3 expressed with or without an N-terminal FLAG-tag induced cell death when cells were stimulated with the necroptotic stimulus, TSI (Fig. 1a); both wild-type RIPK3 constructs phosphorylated MLKL at S358, which is a hallmark of MLKL activation (Fig. 1b). We next immunoprecipitated RIPK3 from *RIPK3*^*−/*−^ HT29 cells via the N-terminal FLAG-tag, and subjected the immunoprecipitates to tryptic digestion, filter-aided sample preparation and analysis by mass spectrometry. Cells were induced with either 2.5 ng/ml doxycycline to reach near-endogenous expression levels, or with 500 ng/mL doxycycline to maximize RIPK3 yield and identification of phosphorylation sites and interactors.

Mass spectrometry of FLAG eluates identified RIPK3 in all samples post-doxycycline induction, and the other core necrosome components, RIPK1 and MLKL, were also observed (Fig. 1c). As expected, the amount of RIPK1 interacting with RIPK3 increased upon TSI treatment, owing to necroptosis pathway activation and necrosome formation. Despite its inability to induce necroptosis upon TSI stimulation, D142N RIPK3 also immunoprecipitated RIPK1, consistent with the idea that RHIM-mediated necrosome formation precedes downstream signaling mediated by RIPK3 kinase activity. Curiously, only D142N, but not wild-type, RIPK3 bound RIPK1 at steady state. The underlying basis is currently unknown, although this result hints that necrosome formation is regulated in part by RIPK3 kinase activity. D142N RIPK3 also completely abolished co-immunoprecipitation of MLKL, consistent with previous reports using co-expressed recombinant proteins [37]. This is likely a consequence of the abolition of RIPK3 autophosphorylation at S227, which mediates essential intermolecular salt bridges with MLKL [37].

While the focus of our study was on the phosphorylation events occurring within RIPK3, mass spectrometry also identified several other proteins that were previously identified to interact with RIPK3 or involved in the TNF-induced necroptotic pathway (Fig. 1c). IKBKG (NEMO), an adaptor protein which binds K63-linked ubiquitination of RIPK1 [46], was observed in the same samples where RIPK1 was observed. FADD was observed in immunoprecipitates of both wild-type and D142N RIPK3 after stimulation with TSI, likely as a result of formation of the cytoplasmic TNF Receptor 1-nucleated complex known as Complex II [47]. Recently, casein kinases were proposed to be components of the necrosome pathway which, depending on context, negated [48] or promoted [49, 50] RIPK3 activation and thus necroptotic signaling. Here, on the other hand, casein kinases were detected in both wild-type and D142N RIPK3 immunoprecipitates, indicating their participation in the necrosome is disconnected from RIPK3 activation. We also observed that FLAG-RIPK3 co-immunoprecipitated several previously uncharacterized putative interactors, with CEMIP and ABCD3 amongst the most enriched hits in cells expressing wild-type RIPK3 post-necroptotic stimulation. We anticipate the interactome data obtained herein could provide a foundation for future studies to identify further regulators of the necroptosis pathway.

### Identification of human RIPK3 phosphorylation sites via mass spectrometry

**Fig. 2:**
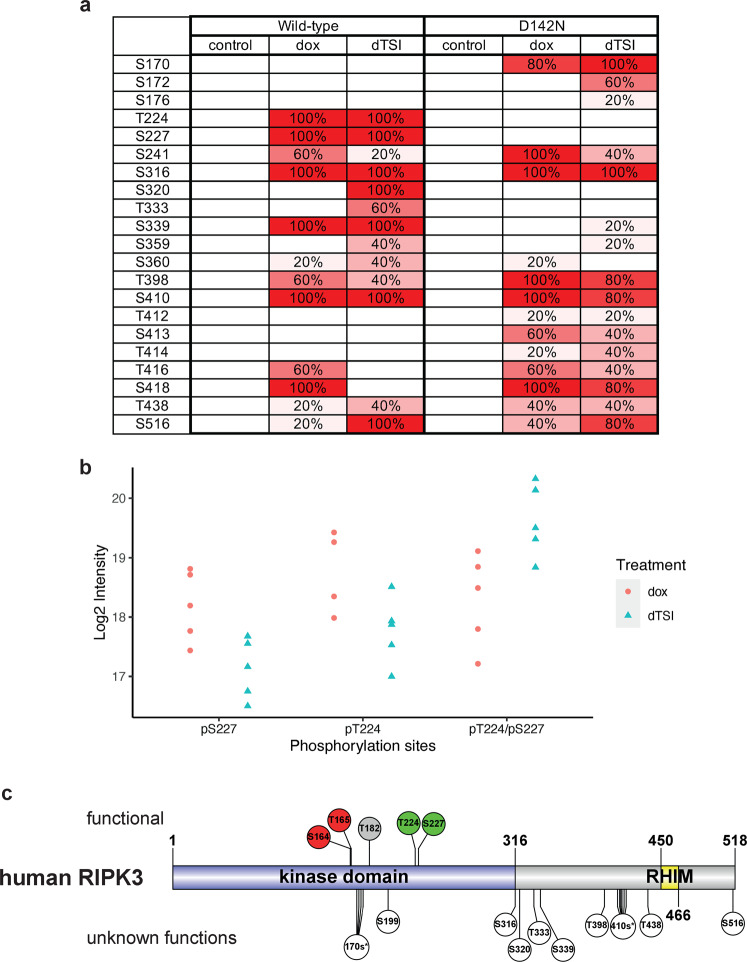
Identification of human RIPK3 phosphorylation sites using mass spectrometry. **a** and **b** RIPK3 phosphorylation sites were detected in the FLAG-immunoprecipitates of HT29 *RIPK3*^−*/*−^ cells containing doxycycline-inducible FLAG-RIPK3 (wild-type and D142N kinase-inactive mutant). Cells were treated with doxycycline (500 ng/mL) overnight, before treatment with dTSI (d, doxycycline; T, TNF; S, Smac mimetic Compound A; I, pan-caspase inhibitor IDN-6556) for 3 h, before lysis and FLAG-immunoprecipitation. **a** Heatmap of the detected RIPK3 phosphorylation sites in cells with wild-type and D142N kinase-inactive mutant FLAG-RIPK3. Numbers indicate the percentage of replicates in each sample in which the phosphorylation was detected (*n* = 5). Color was assigned as a gradient accordingly, where darker red color corresponds to detection of this phosphorylation in more replicates. **b** Scatter plot of log_2_-based peptide intensity values for the pS227 or pT224 single phosphorylation, and pT224/pS227 double phosphorylation tryptic peptide (EVELPTEPSLVYEAVCNR) in *RIPK3*^−*/*−^ HT29 cells with doxycycline-inducible wild-type FLAG-RIPK3, comparing cells stably expressing FLAG-RIPK3 (dox) with those stimulated to undergo necroptosis (dTSI) (*n* = 5). **c** Schematic of human RIPK3 domain architecture and the phosphorylation sites identified. Phosphorylation sites with proposed functions are shown on the top. pT224 and pS227 positively regulates necroptosis (green) by recruiting MLKL. pS164 and pT165 negatively regulate necroptosis by inhibiting RIPK3 kinase activity (red) [38]. Although not detected in our mass spectrometry study, phosphorylation of T182 (grey) was proposed to promote RIPK3 kinase activity and to recruit PELI1 to mediate proteasomal degradation of RIPK3 [54]. Phosphorylation sites with unknown functions are shown on the bottom (white). Asterisks (*) denotes multiple serine/threonine on the same peptide, as such the exact site of phosphorylation could not be unambiguously identified.

We next sought to identify sites of human RIPK3 phosphorylation from our FLAG-immunoprecipitation mass spectrometry data. We treated cells with 500 ng/mL doxycycline to induce robust FLAG-RIPK3 expression in *RIPK3*^*−*^^*/*−^ HT29 cells to allow identification of RIPK3 phosphosites by mass spectrometry, because only a subset of these sites were phosphorylated upon induction of expression with 2.5 ng/mL doxycycline. Among the 21 RIPK3 phosphorylation sites we identified (Fig. 2a), both S227 and T224 in human RIPK3 were phosphorylated in cells expressing wild-type, but not kinase-inactive (D142N), RIPK3, supporting the idea that they are autophosphorylated by RIPK3. Because these are adjacent sites on the same tryptic digested peptide, further analyses revealed that they can indeed be phosphorylated at the same time, and doubly phosphorylated peptides increased slightly upon TSI treatment (Fig. 2b). Similarly, phosphorylation of S320, T333, and S339 was detected predominantly in wild-type, but not D142N, RIPK3, implicating them as autophosphorylation sites. Phosphorylation of RIPK3 S176, S320, and T333 were only detected following TSI stimulation, suggesting their phosphorylation may result from necroptosis signaling. Curiously, in *RIPK3*^−*/*−^ HT29 cells expressing wild-type RIPK3, pT416, pS418 and pT398 abundance decreased upon treatment with TSI, raising the possibility that phosphorylation at these sites could be removed by a cellular phosphatase during necroptotic signaling. Because D142N mutation renders the RIPK3 kinase inactive, phosphorylation sites detected in the C-terminal intermediate region of RIPK3, including S360, T412, S413, T414, T416, S418, T438, and S516 in cells expressing D142N RIPK3 must arise from phosphorylation by kinases other than RIPK3 (Fig. 2c).

### Human RIPK3 T224 and S227 phosphorylation synergistically strengthens interaction with MLKL

**Fig. 3:**
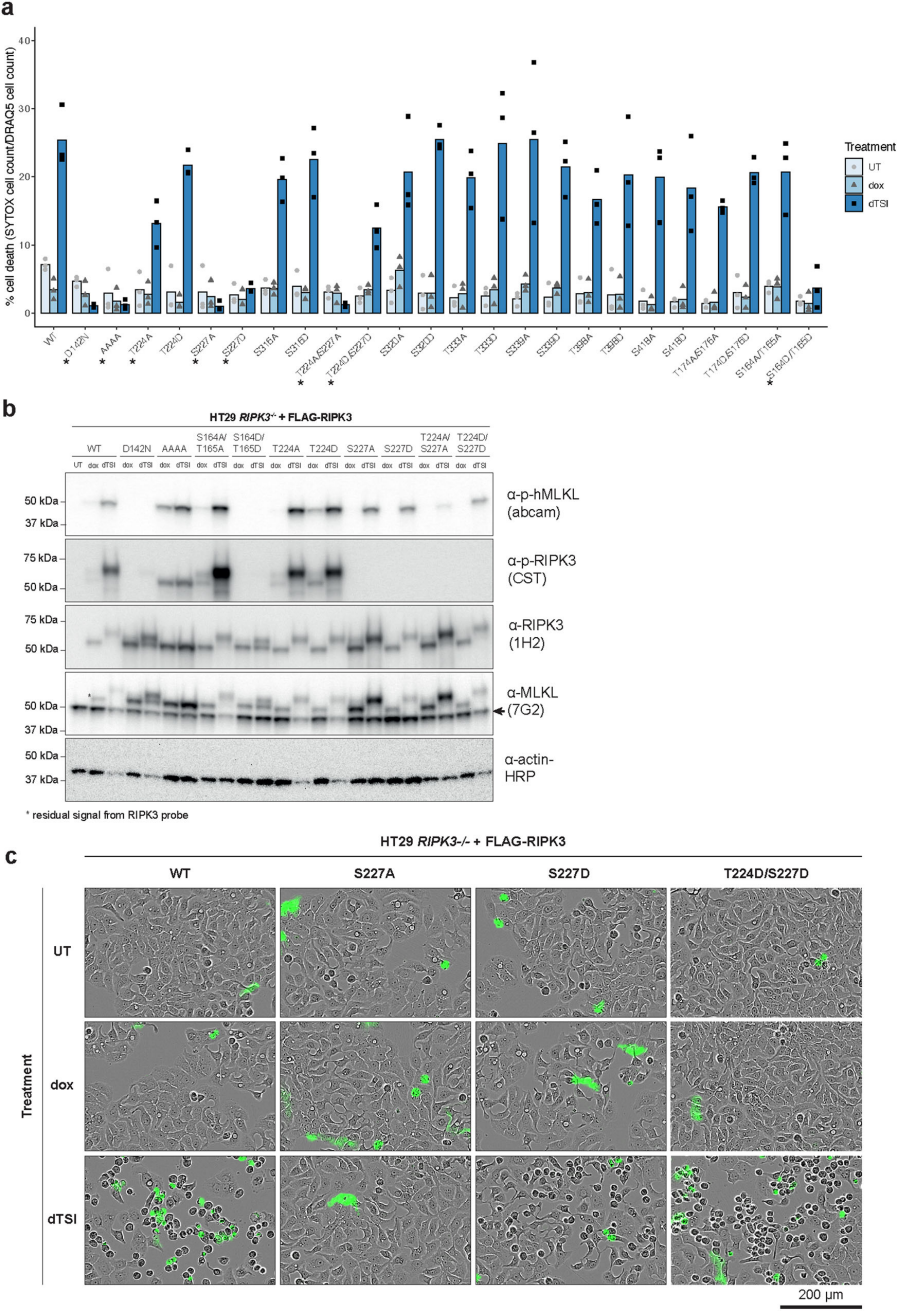
Phosphorylation of kinase domain regulates RIPK3 functions. **a** Necroptotic cell death mediated by wild-type or phosphorylation site mutants of human RIPK3 in *RIPK3*^−*/*−^ HT29 cells. Wild-type (WT) or mutant human RIPK3 expression was induced with 2.5 ng/mL doxycycline (dox) for 16–24 h, and cell death after 24 h of necroptotic stimulation, dTSI (d, doxycycline; T, TNF; S, Smac mimetic Compound A; I, pan-caspase inhibitor IDN-6556), was measured. Cell death (quantified by SYTOX Green uptake) was measured as a percentage of total number of cells (quantified by DRAQ5 uptake) using IncuCyte imaging. The means of three independent experiments (*n* = 3) are displayed as bars. Values of each independent experiments are shown as points. Asterisks (*) indicate RIPK3 mutants that mediated significantly less cell death than the wild-type RIPK3 at 24 h post-necroptotic stimulation (*p* < 0.05 unpaired *t*-test). **b** Evaluation of the abilities of RIPK3 phosphorylation site mutants to mediate MLKL phosphorylation and RIPK3 autophosphorylation in *RIPK3*^−*/*−^ HT29 cells. After being induced with 2.5 ng/mL doxycycline (dox) for 16–24 h, cells expressing wild-type or mutant human RIPK3 were treated with necroptotic stimuli, dTSI, for 7.5 h, before lysis and analysis by immunoblotting with antibodies indicated. Data are representative of three independent experiments (*n* = 3). **c** *RIPK3*^−*/*−^ HT29 cells expressing S227D RIPK3 displayed necroptotic morphology upon dTSI treatment for 24 h, despite failing to uptake SYTOX Green. In contrast, cells with wild-type RIPK3 underwent necroptosis upon dTSI treatment, and cells with T224D/S227D RIPK3 underwent attenuated necroptosis, whereas S227A RIPK3 failed to mediate necroptosis. IncuCyte micrographs taken with the phase channel (greyscale) and green fluorescence channel (green, detect SYTOX Green signal) are overlaid. Data are representative of three independent experiments (*n* = 3).

To examine the biological function of each phosphorylation site identified using mass spectrometry, we substituted each phosphorylation site of interest in FLAG-RIPK3 with either phosphoablating (alanine) or phosphomimetic (aspartate) residues, stably introduced each mutant into *RIPK3*^−*/*−^ HT29 cells (Supplementary Fig. 1), and examined their capacity to reconstitute necroptotic signaling (Fig. 3a, Supplementary Fig. 2a). In addition to mutants of RIPK3 T224, S227, S316, S320, T333, S339, T398, and S418, we also introduced double phosphoablating or phosphomimetic mutations at T224 and S227, because these residues can be phosphorylated at the same time. Because many activation loop phosphorylation sites are on the same peptide, unambiguous identification of each site is difficult. As a result, we introduced T174/S176 and the previously reported S164/S165 [38] double mutants as representative of phosphorylation at different regions of the RIPK3 kinase domain activation loop. The D142N kinase-inactive and RHIM (V^458^QVG > AAAA) RIPK3 mutations were introduced in parallel as negative controls. Following 2.5 ng/mL doxycycline treatment to induce expression levels akin to those of endogenous RIPK3, the ability of each RIPK3 construct to reconstitute necroptotic cell death was examined by IncuCyte live cell imaging (Fig. 3a, Supplementary Fig. 2a). Mutation of each phosphorylation site in the C-terminal intermediate region of human RIPK3 did not affect cell death by necroptosis, although this does not rule out the possibility of roles in other RIPK3-mediated pathways. On the other hand, consistent with a recent report [38], the S164A/T165A RIPK3 mutation reconstituted necroptosis and potently promoted MLKL phosphorylation, but the S164D/T165D phosphomimetic counterpart completely abolished cell death and the ability of RIPK3 to phosphorylate MLKL (Fig. 3b). Upon necroptotic stimulation, wild-type RIPK3 undergoes an upward gel shift (Fig. 3b), which was previously attributed to autophosphorylation at S199 [3], because K50A kinase-inactive and S199A mutants abolished this behavior. However, it is not abolished by D142N kinase-inactive mutant, suggesting that the gel shift is not dependent on autophosphorylation. Curiously, RHIM mutation completely abolished the RIPK3 gel shift, suggesting necrosome formation, or subsequent post-translational modification within the necrosome, may be responsible for this gel shift.

Phosphorylation of both human RIPK3 T224 and S227 contributes to RIPK3-mediated necroptosis, but intriguingly they are not essential for MLKL phosphorylation. The phosphomimetic S227D RIPK3 mutant also failed to reconstitute necroptotic cell death, as measured by SYTOX Green uptake (Fig. 3a), likely due to an aspartate mutation being an imperfect mimic for phosphoserine, which also compromises MLKL binding. Curiously, despite not showing any SYTOX Green uptake, cells expressing S227D RIPK3 exhibited necroptotic morphology upon TSI stimulation, hinting at partial restoration of the necroptotic pathway (Fig. 3c). In contrast, *RIPK3*^−*/*−^ HT29 cells expressing S227A RIPK3 treated with TSI looked identical to untreated cells. Mutation of the adjacent T224 only decreased necroptotic cell death slightly, suggesting that pT224 is less crucial for RIPK3–MLKL interaction than pS227 (Fig. 3a). Although a slight decrease in the ability of T224A RIPK3 to mediate necroptosis was observed previously [37], here, the use of membrane permeable DRAQ5 stain to enumerate total cell numbers and normalize cell death as a percentage of total cell count allowed us to attribute this decrease to abolition of pT224, rather than variability in cell density. Interestingly, when T224D is introduced in concert with the inactivating S227D mutation into human RIPK3, the ability for RIPK3 to reconstitute necroptosis is partially rescued. This demonstrates that phosphorylation of both T224 and S227 are involved in necroptosis signaling, and negative charges at each site act synergistically to mediate RIPK3 function.

Strikingly, all single or double mutants of T224 and S227 retained the ability to phosphorylate MLKL (Fig. 3b), and the occurrence of pMLKL does not correlate with the ability of these mutants to induce necroptotic cell death (Fig. 3a). Rather, it appears that the ability to stably recruit MLKL to RIPK3, or the necrosome, is a more important factor for mediating necroptosis. Despite retaining the ability to phosphorylate MLKL, mutation of RIPK3 S227 abolished necroptotic cell death, as reported previously [2]. The T224D/S227D RIPK3 double mutant could partially reconstitute necroptotic cell death while the S227D mutant could not (Fig. 3a–c), with less pMLKL signal delivered by the T224D/S227D double mutant (Fig. 3b). Even the phosphoablating T224A/S227A RIPK3 double mutant and the RHIM-disruptive RIPK3 mutant were able to phosphorylate MLKL to some extent, despite not promoting necroptotic cell death (Fig. 3b). Similarly, mouse RIPK3 also phosphorylated human MLKL to a small extent, but did not provoke necroptotic cell death (Supplementary Fig. 2b, c), although whether the inability to mediate necroptosis is due to failure to meet a threshold amount of pMLKL or failure to stably recruit MLKL to the necrosome remains to be resolved. Indeed, like the S227A variant of human RIPK3, this finding is consistent with a previous report where it was observed that mouse RIPK3 does not stably bind human MLKL [35]. Collectively, these data illustrate that phosphorylation of MLKL by RIPK3 is necessary but not sufficient for necroptosis. Double phosphorylation of T224 and S227 in RIPK3 works synergistically in the stable recruitment of MLKL. This finding echoes our observations from immunoprecipitation/mass spectrometry (Fig. 1c), where the kinase-inactive D142N mutant RIPK3, which lacks T224 and S227 phosphorylation, did not pulldown MLKL. We propose that this recruitment event occurs upstream of RIPK3-mediated phosphorylation of MLKL in the chronology of necroptotic signaling, where RIPK3 binding enables MLKL to be recruited to the necrosome and connect with other requisite machinery, such as trafficking proteins [27] and chaperones [51, 52], to promote MLKL’s executioner function.

### RIPK3 S227 autophosphorylation at steady state is dependent on MLKL

**Fig. 4:**
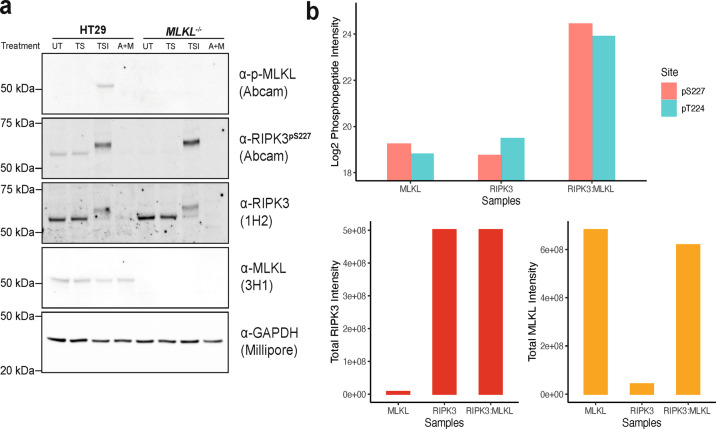
Human RIPK3 S227 autophosphorylation is dependent on the presence of MLKL. **a** Evaluation of endogenous RIPK3 S227 autophosphorylation in parental and *MLKL*^−*/*−^ HT29 cells. Cells were either left untreated (UT), treated with an apoptotic stimulant (TS; T, TNF; S, Smac mimetic Compound A), a necroptotic stimulant (TSI; I, pan-caspase inhibitor IDN-6556), or intrinsic apoptosis stimulant (A + M; A, ABT-737; M, Mcl-1 inhibitor S63485) for 7.5 h, before lysis and analysis by immunoblotting with antibodies indicated. Data are representative of three independent experiments (*n* = 3). **b** Recombinant RIPK3 kinase domain autophosphorylation is upregulated when co-expressed with the MLKL pseudokinase domain in insect cells. Equimolar amounts of purified recombinant RIPK3:MLKL complex, RIPK3 kinase domain expressed alone, and MLKL pseudokinase domain alone were tryptic digested and analyzed by mass spectrometry. *Top*, bar plot of log_2_-based peptide intensity values for tryptic peptide (EVELPTEPSLVYEAVCNR) with phosphorylation of T224 and S227. *Bottom*, bar plot of total RIPK3 (left) or MLKL (right) peptide intensity in each recombinant protein sample.

We sought to further evaluate the role of S227 phosphorylation in RIPK3-mediated signaling. In wild-type HT29 cells, RIPK3 pS227, but not MLKL pS358, was observed under basal conditions and during extrinsic apoptosis (TS; Fig. 4a). By comparison, RIPK3 was degraded during intrinsic apoptosis (A + M; Fig. 4a). As expected, under necroptotic (TSI) conditions, RIPK3 exhibited both a shift in its electrophoretic mobility (previously attributed to S199 phosphorylation [3]) and a marked increase in the phosphorylation of S227. These findings support the idea that phosphorylation of RIPK3 at S227 it is a constitutive event that is increased under necroptotic conditions. Curiously, in *MLKL*^−*/*−^ HT29 cells, phosphorylation of RIPK3 S227 was not detectable under basal conditions (Fig. 4a), but was readily phosphorylated after exposure to a necroptotic stimulus. The constitutive autophosphorylation of RIPK3 S227 under basal conditions is not due to sporadic activation of the necroptosis pathway, because RIPK3 pS227 is also observed in *RIPK1*^−*/*−^ HT29 cells under basal conditions (Supplementary Fig. 3). These data indicate that the autophosphorylation of RIPK3 S227 is MLKL-dependent under resting conditions, but becomes MLKL-independent under necroptotic conditions.

The observation of basally phosphorylated RIPK3 in HT29 cells was unexpected and raised the question whether this could arise from unforeseen behavior of the anti-pS227 RIPK3 antibody. In the *RIPK3*^*−*^^*/*−^ HT29 cell reconstitution studies above (Fig. 3), we monitored pS227 RIPK3 using an antibody from CST, while the Abcam anti-pS227 counterpart was used to monitor endogenous pS227 RIPK3 under basal conditions (Fig. 4). To eliminate the possibility that the unexpected observation of basal pS227 RIPK3 could relate to non-specific antibody binding, we further examined the role of RIPK3 pS227 in MLKL complex formation using recombinant proteins. Previously, we reported the expression and purification of a recombinant protein complex between the human RIPK3 kinase domain and MLKL pseudokinase domain (RIPK3:MLKL herein) [23, 37]. Recombinant RIPK3 and MLKL only form a stable complex when co-expressed in insect cells, because when recombinant RIPK3 and MLKL were purified separately and mixed in vitro, they failed to form a stable complex. Similar behavior had also been reported for the recombinant mouse RIPK3:MLKL complex [36]. We used phosphoproteomics to characterize the phosphorylation status of this complex. Equimolar amounts of purified recombinant RIPK3:MLKL, MLKL pseudokinase domain alone, and RIPK3 kinase domain alone were subjected to tryptic digest and analyses by mass spectrometry (Fig. 4b). Surprisingly, phosphorylation of RIPK3 T224 and S227 was elevated in the RIPK3:MLKL complex in comparison to the RIPK3 kinase domain expressed and purified alone (Fig. 4b). Taken together, these data demonstrate that RIPK3 T224 and S227 autophosphorylation is not only crucial for stable complex formation with MLKL [37], but is also, unexpectedly, dependent on the presence of MLKL.

## Discussion

**Fig. 5:**
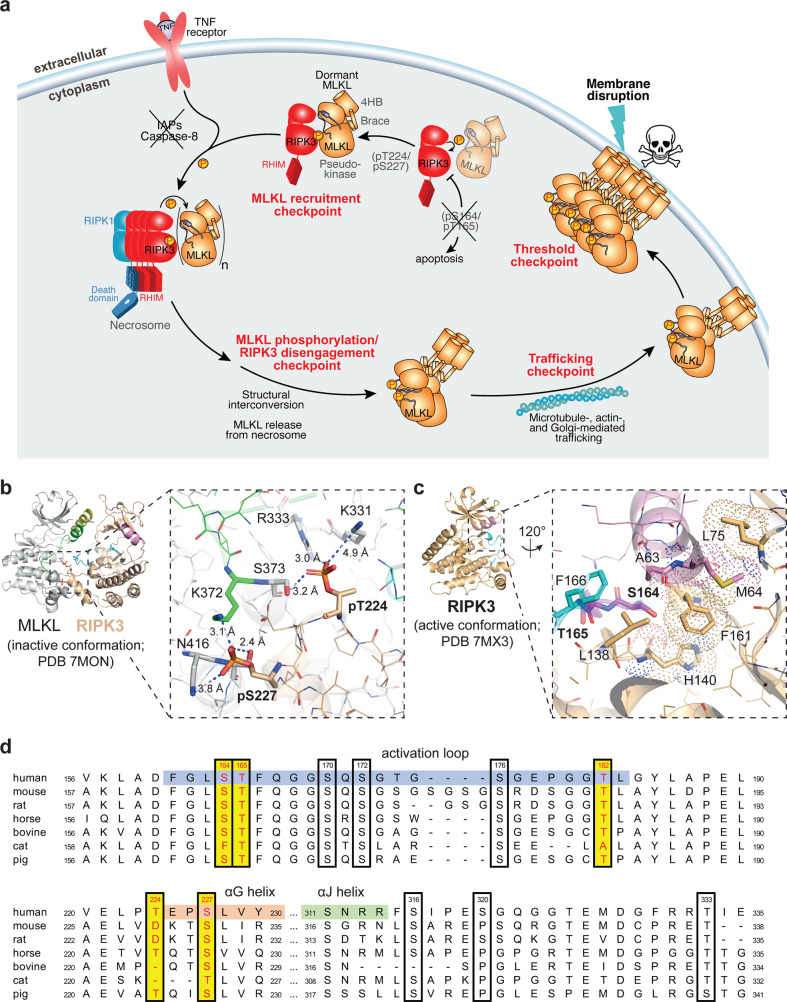
Summary of the regulation of RIPK3 functions by phosphorylation. **a** A schematic diagram of the current model of necroptosis signaling. RIPK3 kinase activity is negatively regulated by pS164/pT165, which promotes apoptotic pathways [38]. Under basal conditions, in the absence of inhibitory pS164/pT165, RIPK3 autophosphorylates T224/S227 in a MLKL-dependent manner, allowing MLKL to be recruited to the kinase domain of RIPK3. Upon TNF signaling in the absence of cIAPs and Caspase-8 activity, MLKL is phosphorylated in the necrosome, before disengaging from the RIPK3 kinase domain, undergoing a structural transition [23, 37], and assembling into active oligomers, which are then trafficked to the plasma membrane, where it accumulates as hotspots to enact cell death [27]. The skull and crossbones motif (Mycomorphbox_Deadly.png; Sven Manguard) is used under a Creative Commons Attribution-Share Alike 4.0 license. **b**, **c** The human RIPK3 crystal structures (wheat; αC helix, pink; activation loop, cyan) rationalize the mechanism by which phosphorylation events regulate RIPK3 functions [37]. Peptide backbones are shown as ribbons. Residues are shown as sticks and colored by chemical element. **b** In the human RIPK3 kinase domain:MLKL pseudokinase domain complex (gray; αC helix, yellow; activation loop, green; PDB 7MON) [37], RIPK3 kinase domain adopts an open, inactive conformation. pT224 and pS227 mediate multiple intermolecular salt bridges and electrostatic interactions (dashes, distance labeled in Å). pT224, pS227, and MLKL-interacting residues are shown as thick sticks. **c** In the closed, active conformer of human RIPK3 kinase domain, S164/T165 reside in a structured region of the activation loop. The side chain of S165 is buried by hydrophobic residues, including the regulatory (R-spine (dots), A63 of αC helix, F166 and L138. S164/T165 and the residues surrounding S164 are shown as thick sticks. **d** Sequence alignment of RIPK3 from model mammalian species (human, *Homo sapiens*, Uniprot Q9Y572; mouse, *Mus musculus*, Uniprot Q9QZL0; rat, *Rattus norvegicus*, Uniprot Q9Z2P5; horse, *Equus caballus*, Uniprot F6SCQ8; bovine, *Bos taurus*, Uniprot E1BKA8; cat, *Felis catus*, Uniprot M3W4D3; pig, *Sus scrofa*, Uniprot F1SGQ6). Only regions with highly conserved phosphorylation sites are shown. Phosphorylation sites identified in this study are shown with black box with residue number (according to human RIPK3 sequence) labeled on top. Phosphorylation sites with proposed roles in necroptotic signaling are colored red with a yellow background. Relevant secondary structures are highlighted on the human RIPK3 sequence according to the crystal structure of human RIPK3 kinase domain (shown in **b**; PDB 7MX3) [37]. Alignment was performed using Clustal Omega algorithm in Snapgene.

The necroptosis pathway relies on a cascade of post-translational modifications [30], which include the autophosphorylation of RIPK1 and RIPK3, and RIPK3-mediated phosphorylation of MLKL. However, the precise mechanism by which necroptotic signals are relayed between the necrosome components is incompletely understood, and recent studies have called into question whether pS227 is a sufficient cue for RIPK3 activation [23, 37]. Here, phosphoproteomics allowed us to identify a total of 21 phosphorylation sites in human RIPK3, although only pT224 and pS227, in addition to the recently described pT164/S165 [38], were found to serve crucial roles in necroptosis from mutational studies. Our data indicate that while pT224 acts synergistically with pS227 to stably recruit MLKL to the necrosome, unexpectedly, neither pT224 nor pS227 are required to phosphorylate MLKL, and pMLKL levels do not correlate with the ability of cells to undergo necroptosis. Instead, the ability of RIPK3 to stably bind MLKL is a more important factor in mediating necroptotic cell death, because MLKL was able to be phosphorylated by RIPK3 variants carrying T224, S227, or RHIM mutation, but failed to induce necroptosis. These results mirror earlier findings where the V220E, L222D and A232R RIPK3 mutants disrupted the RIPK3:MLKL interface and abolished cell death, while retaining their capacity to phosphorylate MLKL [37]. We propose that the stable recruitment of MLKL to the necrosome is an essential necroptotic checkpoint, which is independent from the phosphorylation of MLKL (Fig. 5a). Furthermore, our data raise the possibility that enhanced RIPK3 S227 phosphorylation, rather than basal pS227 and additional phosphorylation events in RIPK3, are the critical cues for RIPK3 activation and/or the formation of a stable necrosome prior to necroptotic death. Our data do not exclude the possibility of other post-translational modifications or protein interactions as the trigger for RIPK3 recruitment to the necrosome and activating phosphorylation of MLKL.

Since the discovery of MLKL as the terminal effector of necroptosis, phosphorylation of RIPK3 S227 was known to be essential for MLKL interaction and necroptotic cell death [2]. Our data support the idea that pS227 is essential for MLKL recruitment, which is augmented by the adjacent pT224 via synergistic intermolecular salt bridges [37] (Fig. 5b). Although it was initially presumed that pS227 is the cue for RIPK3 activation [2], which functions by recruiting and allowing RIPK3 kinase domain to phosphorylate MLKL, our data suggest that the recruitment of MLKL to the necrosome and the phosphorylation of MLKL are two independent checkpoints, because all T224 and S227 RIPK3 mutants retained the ability to phosphorylate MLKL, despite having attenuated or abolished cell death functions, and the ability to partially restore necroptosis signaling correlates with the ability of RIPK3 mutants to stably interact with MLKL. These mutants phenocopy mouse RIPK3, which is also unable to stably interact with human MLKL [35] or mediate cell death in human cells but retains partial ability to phosphorylate human MLKL. The purpose of the RIPK3:MLKL stable interaction is likely the recruitment of MLKL to the necrosome, because abolishing necrosome assembly via RIPK3 RHIM mutation also resulted in constitutive phosphorylation of MLKL without any cell death, and MLKL activated by forced dimerization cannot induce cell death in absence of RIPK1 [53]. It is likely that like RIPK1, RIPK3 also serves scaffolding functions downstream of MLKL phosphorylation, because the recruitment of MLKL to the necrosome may be necessary for MLKL to engage with downstream modulators, such as trafficking partners [27] or chaperones [51, 52].

Recent studies of the endogenous human RIPK3:MLKL interaction support that idea that the recruitment of MLKL to RIPK3, or the necrosome, precedes the phosphorylation of MLKL [23, 37]. A synthetic protein ligand, Mb27, which binds MLKL via an overlapping interface with RIPK3, failed to bind endogenous MLKL under basal conditions, suggesting that MLKL resides in stable complex with RIPK3 at steady state. Subsequently, the crystal structure of RIPK3:MLKL complex revealed that this stable assembly is mediated by pS227 and pT224, and pS227 is essential for RIPK3:MLKL complex formation [37]. In keeping with their obligate role in MLKL engagement, in this work, we detected pS227 in endogenous RIPK3 in HT29 cells under basal conditions. Interestingly, the phosphorylation of RIPK3 S227 at steady state is dependent on the presence of MLKL, both in cells and in recombinant complexes. This raises the possibility that MLKL allosterically activates RIPK3 to undergo autophosphorylation, or that MLKL binding precludes a cellular phosphatase from hydrolyzing RIPK3 pS227 under steady state.

In keeping with the proposed pro-apoptotic role of pS164/pT165 [38], phosphorylation at these sites were not detected in HT29 cells under necroptotic conditions. However, we were able to verify that phosphomimetic mutation of these residues potently inhibited RIPK3 kinase activity, and abolished RIPK3 autophosphorylation and MLKL phosphorylation. The mechanism of such potent inhibition can be rationalized from reported crystal structures of human RIPK3 [37] (Fig. 5c). S164 and T165 reside on the activation loop of RIPK3 kinase domain, which is disordered when RIPK3 kinase is in the inactive conformation. However, in the active RIPK3 conformation, the activation loop is structured and S164 is buried within a hydrophobic pocket between the aligned R-spine residues, A63 (αC helix), L138, and F166. As such, introduction of a negatively charged, bulky phosphate group at S164 would severely disrupt the active conformer of human RIPK3 in favor of the inactive conformation where pS164 is solvent exposed. This result also supports the observation that the human RIPK3 conformation captured in complex with MLKL is kinase inactive [37].

All three clusters of phosphorylation sites that have been proposed to regulate RIPK3 functions, pT224/pS227, pS164/pT165 [38], and pT182 [54], reside in the C-lobe of the kinase domain. Interestingly, we did not detect T182, S164, or T165 phosphorylation, nor the previously reported interaction with PELI1 [54], in our study, raising the prospect that this interaction is cell and context specific. With the exception of feline RIPK3, S227, S164/T165, and T182 are well conserved among mammals (Fig. 5d). T224 of human RIPK3, however, is only conserved in horse and pig, but not the murine ortholog. This likely has contributed to the species-specificity of the RIPK3:MLKL pair, such that necroptosis in human cells can be reconstituted with porcine MLKL but not murine MLKL [55]. Amongst other human RIPK3 phosphorylation sites identified in this study, we found that S170, S172, and S176 in the kinase domain activation loop, as well as S316 and T333, in a disordered region immediately following the kinase domain, are highly conserved across mammalian species. Although we have not identified a regulatory role of these sites in necroptosis, it is possible that phosphorylation at these sites could regulate other RIPK3-mediated functions.

In the present work, we defined the synergistic role of RIPK3 pT224 and pS227 in stably recruiting MLKL, which is independent from the phosphorylation of MLKL. We propose that RIPK3-mediated recruitment of MLKL to the necrosome is an upstream necroptosis checkpoint, which occurs prior to the phosphorylation of MLKL following a cell’s exposure to a necroptotic stimulus. Our data reveal additional RIPK3 autophosphorylation sites and sites phosphorylated by other kinases, in addition to RIPK3 interactors, which could aid future studies to uncover additional regulatory mechanisms of RIPK3-mediated pathways.

## Supplementary information

Supplementary Figures 1-3

Author checklist

## Data Availability

All data, including expression construct sequences, are available from the corresponding authors upon request. The mass spectrometry proteomics data have been deposited to the ProteomeXchange Consortium via the PRIDE partner repository with the dataset identifier PXD033488.
