# Supplementary material for: Human RIPK3 C-lobe phosphorylation is essential for necroptotic signaling
Source: Cell Death Dis. 2022 Jun 23;13(6):565. doi: 10.1038/s41419-022-05009-y (PMC9226014; doi:10.1038/s41419-022-05009-y)

SUPPLEMENTARY FIGURES

Supplementary Figure 1. Expression of N-FLAG human RIPK3 constructs in HT29 *RIPK3$^{-/-}$* cells. Expression of wild-type and phosphorylation site RIPK3 mutants upon overnight doxycycline treatment (2.5 ng/ml). Immunoblots are representative of 3 independent experiments.

Supplementary Figure 1

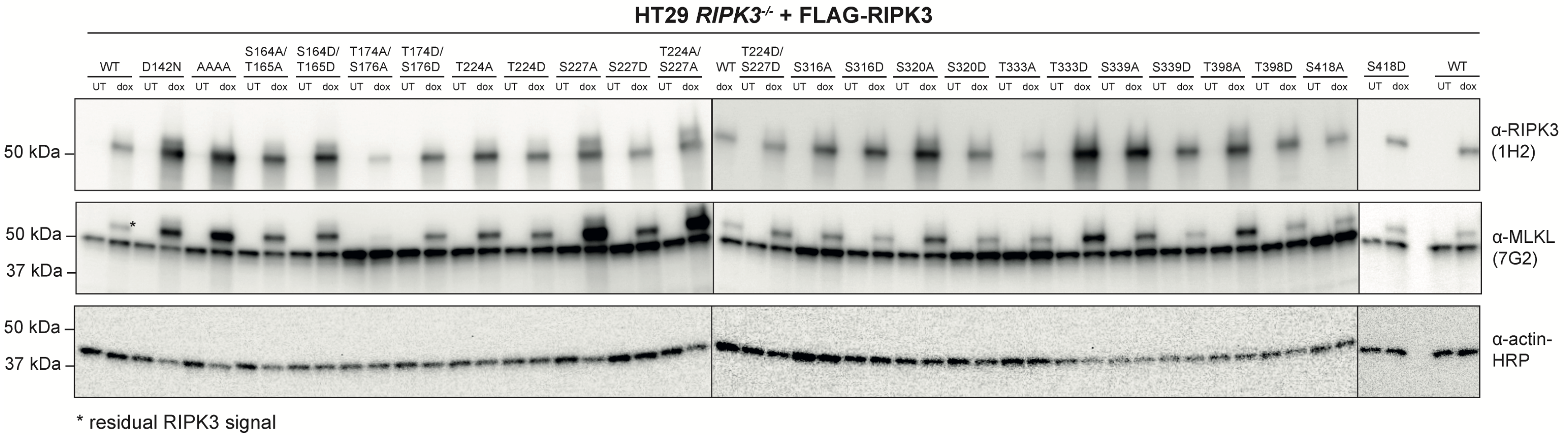

Supplementary Figure 2. Necroptotic cell death mediated by RIPK3 phosphorylation site mutants in HT29 *RIPK3$^{-/-}$* cells. a-b. RIPK3 expression was induced with doxycycline (2.5 ng/ml) for 16-24 hours, before cells were treated with the necroptotic stimulus, dTSI (d, doxycycline; T, TNF; S, Smac mimetic Compound A; I, pan-caspase inhibitor IDN-6556). Measurements at individual time points are shown as crosses. Mean of three independent experiments (n=3) is shown as line plot. Red, cells treated with dTSI; black, cells treated with doxycycline. **a.** Cell death induced by the expression of each N-FLAG human RIPK3 construct was monitored over the course of 24 hours since the addition of dTSI using IncuCyte SX5 (Sartorius). Cell death (quantified by SYTOX Green uptake) was quantified as a percentage of total number of cells (quantified by DRAQ5 uptake). **b**. Mouse RIPK3 does not mediate necroptosis in human *RIPK3$^{-/-}$* HT29 cells, despite being able to phosphorylate MLKL. Cell death was quantified as number of SYTOX Green-positive cells per mm^2 using IncuCyte S3 imaging over the course of 24 hours. Mean of three independent experiments (n=3) is shown as a line plot, except for the measurements at 0-6 hours where data are missing for one replicate due to an acquisition error (n=2 for measurements at 0-6 hours). **c.** After being induced with 2.5 ng/ml doxycycline (dox) for 16-24 hours, cells expressing wild-type or D142N kinase-inactive human RIPK3 or mouse RIPK3 were treated with necroptotic stimuli, dTSI, for 7.5 hours, before lysis and analysed by immunoblotting with antibodies indicated. Data are representative of three independent experiments (n=3).

Supplementary Figure 2

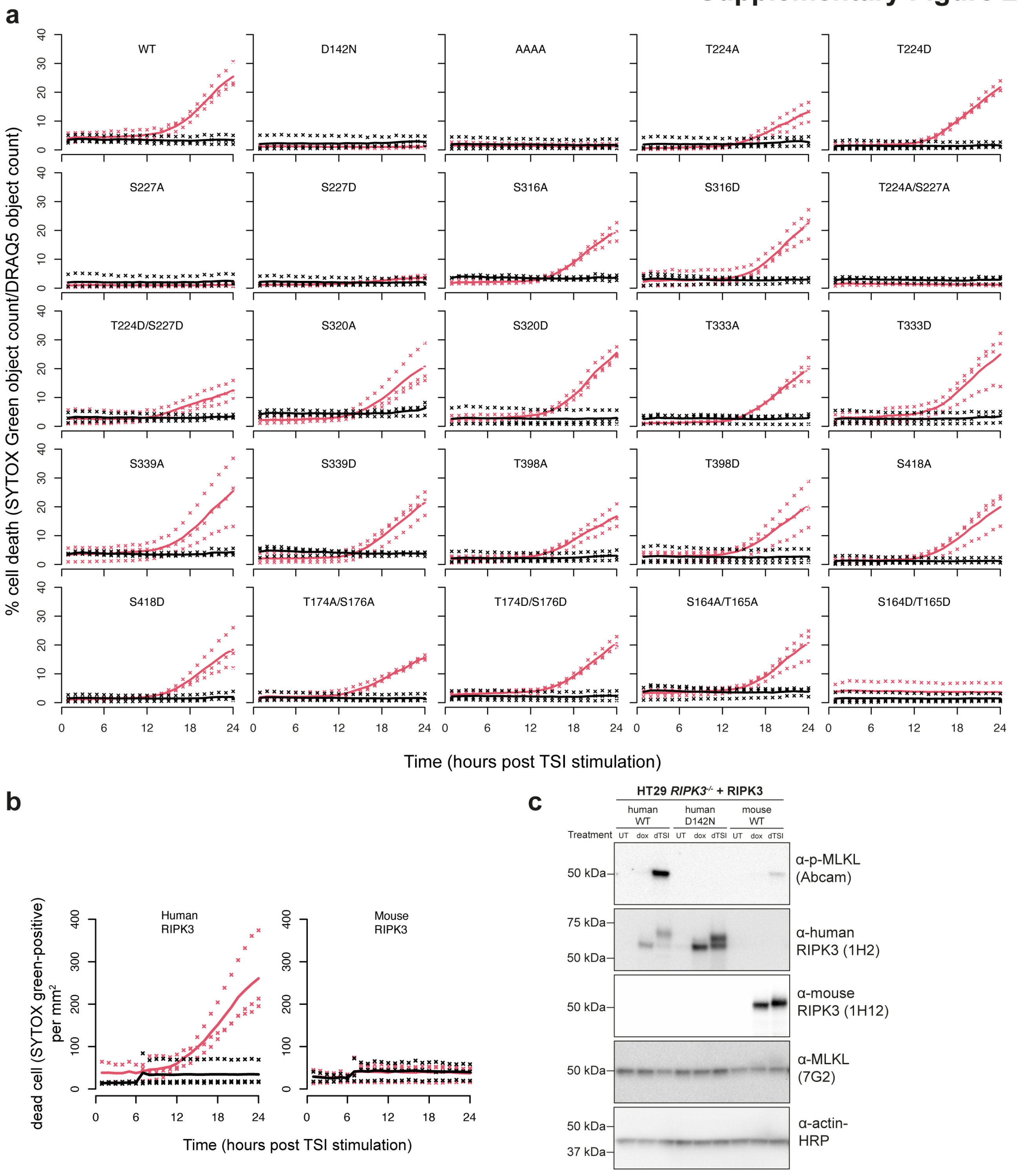

Supplementary Figure 3. Human RIPK3 autophosphorylation in the absence of necroptotic stimulation is independent from RIPK1. Unstimulated parental HT29 cells and their *RIPK1*$^{-/-}$, *RIPK3*$^{-/-}$ or *MLKL*$^{-/-}$ counterparts were analyzed by immunoblotting with the indicated antibodies. Arrows indicate full-length, uncleaved species.

Supplementary Figure 3

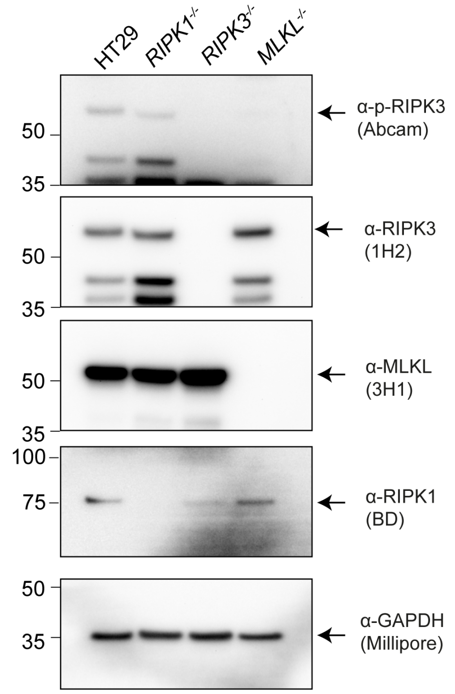

Supplement: Supplementary file 1 — Supplementary Figures 1-3 [file 41419_2022_5009_MOESM1_ESM.pdf]
